# Establishment of a nomogram model for predicting adverse outcomes in advanced-age pregnant women with preterm preeclampsia

**DOI:** 10.1186/s12884-022-04537-x

**Published:** 2022-03-19

**Authors:** Bohan Lv, Yan Zhang, Guanghui Yuan, Ruting Gu, Jingyuan Wang, Yujiao Zou, Lili Wei

**Affiliations:** 1 School of Nursing, Qingdao University, Qingdao, China; 2 Department of Nursing, The Affiliated Hospital of Qingdao University, #16 Jiangsu Road, Qingdao, 266003 Shandong Province China; 3 Medical College of Qingdao University, Qingdao, China; 4 Department of Respiratory and Critical Care Medicine, The Affiliated Hospital of Qingdao University, Qingdao, China

**Keywords:** Preterm preeclampsia, Advanced age pregnant women, Nomogram, Prediction model

## Abstract

**Aim:**

To establish a model for predicting adverse outcomes in advanced-age pregnant women with preterm preeclampsia in China.

**Methods:**

We retrospectively collected the medical records of 896 pregnant women with preterm preeclampsia who were older than 35 years and delivered at the Affiliated Hospital of Qingdao University from June 2018 to December 2020. The pregnant women were divided into an adverse outcome group and a non-adverse outcome group according to the occurrence of adverse outcomes. The data were divided into a training set and a verification set at a ratio of 8:2. A nomogram model was developed according to a binary logistic regression model created to predict the adverse outcomes in advanced-age pregnant women with preterm preeclampsia. ROC curves and their AUCs were used to evaluate the predictive ability of the model. The model was internally verified by using 1000 bootstrap samples, and a calibration diagram was drawn.

**Results:**

Binary logistic regression analysis showed that platelet count (PLT), uric acid (UA), blood urea nitrogen (BUN), prothrombin time (PT), and lactate dehydrogenase (LDH) were the factors that independently influenced adverse outcomes (*P* < 0.05). The AUCs of the internal and external verification of the model were 0.788 (*95% CI*: 0.737 ~ 0.764) and 0.742 (*95% CI*: 0.565 ~ 0.847), respectively. The calibration curve was close to the diagonal.

**Conclusions:**

The model we constructed can accurately predict the risk of adverse outcomes of pregnant women of advanced age with preterm preeclampsia, providing corresponding guidance and serving as a basis for preventing adverse outcomes and improving clinical treatment and maternal and infant prognosis.

## Background

Preeclampsia (PE) is the main cause of maternal and perinatal death, accounting for 14% of total maternal deaths [1–3]. In China, the incidence of PE is between 2 and 7% [4]. Among pregnant women, PE damages the liver, kidney and blood coagulation system. If disease progression is not detected in time, eclampsia, pulmonary oedema, cerebrovascular accident and even death will occur with high probability [5–7]. For foetuses, PE increases the probability of stillbirth, premature birth, and very low birth weight. Moreover, the risk of long-term complications, such as neurodevelopmental disorders, insulin resistance, diabetes, coronary heart disease and hypertension, is elevated [8–11].

At present, there is no effective treatment for preeclampsia. Expecting treatment and timely delivery according to the specific patient remains the best course of action. For pregnant women with early-onset preeclampsia (gestational age of onset less than 34 weeks), timely delivery or termination of pregnancy is protective for both the mother and infant. However, they may also both experience various adverse outcomes, such as eclampsia, HELLP syndrome, neonatal respiratory distress syndrome, retinopathy of prematurity, and neonatal thrombocytopenia [12, 13]. There are many reasons for a poor outcome, among which greater age is an important factor. With increasing age, a pregnant woman with preeclampsia is more likely to have adverse outcomes [14].

Advanced-age pregnancy is defined as pregnancy in women aged ≥35 years at the expected delivery date, and advanced age itself is associated with a high-risk pregnancy [15]. With the issuance of China’s “three-child policy”, the problem of advanced-age pregnancy has received increasing attention. PE in advanced-age pregnant women is complex and serious, significantly increasing the probability of an adverse pregnancy outcome. Older women with preeclampsia are prone to eclampsia, preterm delivery, and even perinatal death and other adverse outcomes. Therefore, focusing on advanced-age pregnant women with preeclampsia and predicting their adverse outcomes will have important clinical implications for prevention of these outcomes and the reduction of harm to the mother and infant.

At present, there are many models for predicting adverse outcome in patients with PE, the most famous of which is the fullPIERS model established by von Dadelszen et al. in 2011 [16]. However, due to differences between regions and races, there may be limits on the promotion of this model in China. Additionally, the model does not address the special group of advanced-age pregnant women with PE. Furthermore, there are still some prediction models [17, 18] that have not been applied in clinical practice in China because of a lack of external validation, complexity, difficulty obtaining prediction indicators and so on. The purpose of this study was to establish a nomogram model to predict the adverse outcomes of pregnant women of advanced age with preterm PE in China. This will not only help draw researchers’ attention to this special group but also help improve the specificity and accuracy of adverse outcome prediction for this group.

## Methods

### Study design

This was a retrospective study that was approved by the Ethics Committee of the Affiliated Hospital of Qingdao University. Because of the retrospective nature of the study, informed patient consent was waived by the Ethics Committee of the Affiliated Hospital of Qingdao University.

### Participants

We reviewed all the medical records of pregnant women with PE who underwent prenatal examinations and delivery in the obstetrics department of the Affiliated Hospital of Qingdao University from June 2018 to December 2020. Women were included if they had PE or developed PE after admission. Those with gestational age ≥ 37 weeks, twin pregnancy, foetal malformation, age < 35 years old, cardiovascular disease, immune disease or vascular disease were excluded. According to the International Society for the Study of Hypertension in Pregnancy (ISSHP) in 2014 [19], PE was diagnosed as gestational hypertension and the coexistence of one or more of the following new-onset conditions: Proteinuria (spot urine protein/creatinine> 30 mg/mmol [0.3 mg/mg] or > 300 mg/day or at least 1 g/L[‘2+’] on dipstick testing), other maternal organ dysfunction (including renal insufficiency, liver involvement, neurological complications, haematological complications), or uteroplacental dysfunction (foetal growth restriction).

### Variables included for analysis

All of the following data were obtained from the maternal medical records: (1) Demographic variables: age, number of pregnancies, number of births, body mass index (BMI), family history of cardiovascular disease, past history of hypertension, past history of eclampsia or PE, irregular menstruation before pregnancy; (2) clinical symptoms: headache, dizziness, oedema of the lower limbs, blurred vision, and maximum blood pressure; (3) laboratory indices: neutrophil count (NC), monocyte count (MC), lymphocyte count (LC), white blood cell count (WBC), red blood cell count (RBC), haemoglobin (HGB), platelet count (PLT), D-dimer, prothrombin time (PT), prothrombin time activity (PTA), thrombin time (TT), alanine aminotransferase (ALT), aspartate aminotransferase (AST), uric acid (UA), triglyceride (TG), total cholesterol (TCHO), low-density lipoprotein (LDL), high-density lipoprotein (HDL), lactate dehydrogenase (LDH), blood urea nitrogen (BUN), biparietal diameter (BPD), head circumference (HC), femur length (FL), amniotic fluid index (AFI), foetal heart rate (FHR), umbilical artery blood velocity (S/D), pulsatility index (PI), and resistance index (RI). All the included indicators were determined based on a large literature review and the specific hospital medical record system.

### Outcomes

The primary outcome index was adverse outcome, defined based on the 2009 World Health Organization standards for critical maternal illness and the standards used in the fullPIERS model built by von Dadelszen et al. in 2011 [16, 20]. Pregnant women with one or more of the following conditions were considered to have an adverse outcome: eclampsia, HELLP syndrome, cerebrovascular accident, placental abruption, heart failure, pulmonary oedema, detached retina, postpartum haemorrhage, disseminated intravascular coagulation, maternal mortality, hepatic injury, acute kidney injury and foetal death due to PE.

### Statistical analysis

Continuous variables are presented as X (mean) ± SD (standard deviation) or the median and interquartile range. Categorical data are presented as percentages (%). The data were divided into a training set and a verification set at a ratio of 8:2. The model was trained with the training set data and verified with the verification set data. To determine the risk factors for adverse outcomes in advanced-age pregnant women with preterm PE, we first used univariate analysis for preliminary screening, in which continuous variables were tested by the t-test and rank-sum test, and categorical variables were analysed by the chi-square test. Then, we performed collinearity analysis and multifactor logistic regression for the obtained factors (*P* < 0.05 in univariate analysis) and finally determined the relevant independent risk factors (*P* < 0.05). Finally, a nomogram model was drawn according to the multifactor logistic regression model to predict the occurrence of adverse outcomes in pregnant women of advanced age with preterm PE.

In this study, ROC curves and the AUCs were used to evaluate the model fit. A total of 1000 bootstrap samples were drawn from the training set and used for internal verification. Additionally, a calibration chart was drawn to evaluate the difference between the occurrence of adverse outcomes predicted by the prediction model and the actual occurrence in the data. Finally, the model was externally validated with the verification set.

## Results

### Univariate analysis for adverse outcomes

A total of 896 subjects were included in this study and divided into an adverse outcome group and a non-adverse outcome group according to the occurrence of adverse outcomes. There were 133 subjects in the adverse outcome group (14.84%). The details of the patients are shown in Table 1. Certain patients had multiple diseases. In addition, adverse outcomes in newborns included: Low birth weight (82,9.15%), Asphyxia Neonatorum (41,4.57%), neonatal intracranial hemorrhage (3,0.33%), Neonatal infection (5,0.56%). As we focused on the adverse outcomes of pregnant women, the adverse outcomes of newborns were not included in the scope of the adverse outcome referred to in this study. The subjects were divided into two groups at a ratio of 8:2. A total of 110 (15.09%) of the 729 subjects in the training set had adverse outcomes, and 23 (13.77%) of the 167 subjects in the verification set had adverse outcomes. Univariate analysis of the data showed that diastolic blood pressure, neutrophil count, white blood cell count, platelet count, prothrombin time, prothrombin time activity, thrombin time, ALT, AST, UA, LDH, BUN, S/D, PI, and RI were influencing factors of adverse outcomes (Table 2).

**Table 1:** Adverse outcomes of advanced-age pregnant women with preterm preeclampsia

Types	Number
Eclampsia	4 (3.01%)
HELLP syndrome	47 (35.34%)
Cerebrovascular accident	2 (1.50%)
Placental abruption	53 (39.85%)
Heart failure	2 (1.5%)
Retinal detachment	1 (0.75%)
Postpartum haemorrhage	48 (36.09%)
DIC	3 (2.26%)
Foetal death	13 (9.78%)
Hepatic injury	1 (0.75%)
Acute kidney injury	5 (3.76%)

**Table 2:** Comparison of clinical data between the adverse outcomes and non-adverse outcomes groups in the training set (*N* = 729)

Variables	No adverse outcomes ***n*** = 619	adverse outcomes ***n*** = 110	***P***
Age, years	38.36 ± 2.64	38.33 ± 2.89	0.940
Number of pregnancies	3 [2,4]	3 [2,4]	0.435
Number of births	1 [1,1]	1 [1,1]	0.454
Systolic blood pressure, mmHg	158.43 ± 23.52	162.92 ± 23.17	0.065
Diastolic blood pressure, mmHg	98.44 ± 15.98	102.08 ± 15.39	0.027
Body mass index (BMI), kg/m^2^	30.27 ± 3.02	30.09 ± 2.50	0.521
Family history of cardiovascular disease	172 (27.8%)	31 (28.2%)	0.932
Past history			
Hypertension	125 (20.2%)	21 (19.1%)	0.790
Eclampsia or PE	22 (3.6%)	4 (3.6%)	0.966
Irregular menstruation before pregnancy	70 (11.3%)	15 (13.6%)	0.483
Symptoms			
Headache	117 (18.9%)	21 (19.1%)	0.963
Dizzy	106 (17.1%)	21 (19.1%)	0.616
Oedema of the lower limbs	87 (14.1%)	16 (14.5)	0.892
Blurred vision	58 (9.4%)	13 (11.8%)	0.425
Neutrophil count (NC), 10^9^/L	9.05 ± 4.16	9.05 ± 4.16	0.010
Monocyte count (MC), 10^9^/L	0.59 ± 0.23	0.62 ± 0.44	0.372
Lymphocyte count (LC), 10^9^/L	1.81 ± 0.58	1.71 ± 0.60	0.102
White blood cell count (WBC), 10^9^/L	10.22 ± 3.06	11.46 ± 4.43	0.013
Red blood cell count (RBC), 10^12^/L	4.12 ± 0.47	4.06 ± 0.57	0.270
Haemoglobin (HGB), g/L	121.03 ± 15.55	120.67 ± 17.08	0.824
Platelet count (PLT), 10^9^/L	203.11 ± 62.52	171.98 ± 64.41	< 0.001
D-dimer, mg/L	4.32 ± 1.21	4.21 ± 1.32	0.821
Prothrombin time (PT), seconds	9.60 [8.90,10.11]	10.07 [9.17,10.50]	< 0.001
Prothrombin time activity (PTA), %	142.00 [133.89,158.00]	137.29 [129.17,151.00]	0.009
Thrombin time (TT), Seconds	14.76 [13.40,15.66]	15.81 [13.80,16.50]	< 0.001
Alanine aminotransferase (ALT), U/L	23.06 ± 14.22	25.69 ± 10.50	< 0.001
Aspartate aminotransferase (AST), U/L	24.49 ± 13.09	30.52 ± 12.93	< 0.001
Uric acid (UA), μmol/L	372.72 ± 81.15	412.19 ± 70.47	< 0.001
Triglyceride (TG), mmol/L	3.34 ± 1.26	3.29 ± 1.09	0.671
Total cholesterol (TCHO), mmol/L	6.11 ± 1.46	6.11 ± 1.41	0.749
Low-density lipoprotein (LDL), mmol/L	3.16 ± 0.83	3.20 ± 0.82	0.645
High-density lipoprotein (HDL), mmol/L	1.97 ± 0.39	1.94 ± 0.44	0.499
Lactate dehydrogenase (LDH), U/L	234.54 ± 68.21	290.87 ± 77.37	< 0.001
Blood urea nitrogen (BUN), mmol/L	4.66 ± 1.26	5.51 ± 1.46	< 0.001
Biparietal diameter (BPD), cm	7.78 ± 1.16	7.83 ± 0.94	0.772
Head circumference (HC), cm	28.62 ± 2.97	28.64 ± 2.40	0.632
Femur length (FL), cm	5.68 ± 1.04	5.73 ± 0.90	0.726
Amniotic fluid index (AFI), cm	11.65 ± 2.81	12.04 ± 2.86	0.081
Foetal heart rate (FHR), beats/minute	143.69 ± 8.18	143.75 ± 8.83	0.940
Umbilical artery blood velocity (S/D)	2.76 ± 0.46	2.84 ± 0.37	< 0.001
Pulsatility index (PI)	1.04 ± 0.21	1.10 ± 0.21	0.004
Resistance index (RI)	0.62 ± 0.06	0.63 ± 0.05	0.004

### Binary logistic regression analysis for adverse outcomes

According to the collinearity analysis of the influencing factors obtained in the univariate analysis, white blood cell count and neutrophil count had a VIF ≥ 10. According to previous studies [21], white blood cell count and PE was more correlated comparing to neutrophil count. The white blood cell count was selected. A binary logistic regression model was established with adverse outcomes as the dependent variable and the above residual variables as the covariates. The results revealed platelet count (PLT), uric acid (UA), blood urea nitrogen (BUN), prothrombin time (PT), and lactate dehydrogenase (LDH) as independent influencing factors of adverse outcome in preeclampsia (Table 3).

**Table 3:** Binary logistic regression analysis of adverse outcomes in advanced-age pregnant women with preterm PE (N = 729)

Variables	***B***	***SE***	***OR (95%CI)***	***P***
Platelet count (PLT)	−0.004	0.002	0.996 (0.992–0.999)	0.016
Uric acid (UA)	0.003	0.002	1.003 (1.001–1.006)	0.041
Blood urea nitrogen (BUN)	0.340	0.091	1.405 (1.176–1.677)	< 0.001
Prothrombin time (PT)	0.369	0.092	1.446 (1.206–1.732)	< 0.001
Lactate dehydrogenase (LDH)	0.007	0.001	1.007 (1.004–1.010)	< 0.001

^*^B = regression coefficient; *SE =* standard error

### Nomogram and evaluation of the adverse outcome prediction model

**Fig. 1:**
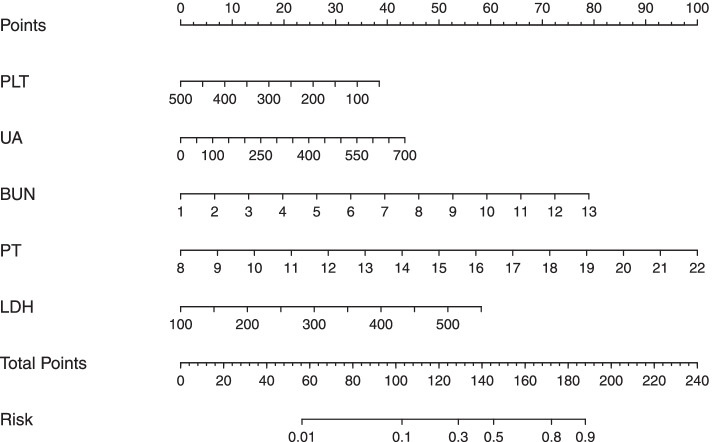
Nomogram model for predicting the risk of adverse outcomes in advanced-age pregnant women with preterm preeclampsia. PLT platelet count; UA uric acid; BUN blood urea nitrogen

**Fig. 2:**
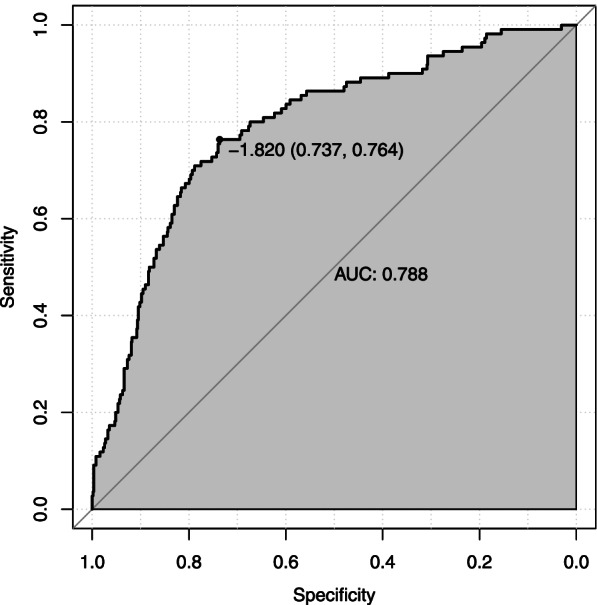
ROC curve of the nomogram model with the training set

**Fig. 3:**
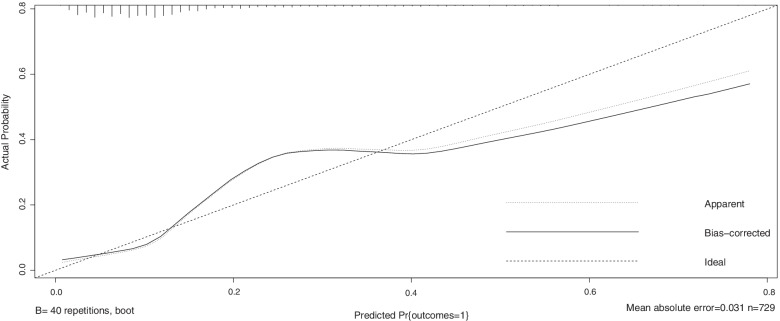
Calibration curve of the nomogram model with the training set

According to the above data analysis, we developed a nomograph prediction model with the 5 independent indices, including platelet count (PLT), uric acid (UA), blood urea nitrogen (BUN), prothrombin time (PT) and lactate dehydrogenase (LDH) (Fig. 1). The AUC of the model was 0.788 (*95%CI*: 0.737 ~ 0.764) (Fig. 2). The nomogram model was internally validated with 1000 bootstrap samples. The calibration curve shows the possibility of using the nomogram model to predict the actual probability of adverse outcomes in advanced-age pregnant women with preterm PE (Fig. 3).

**Fig. 4:**
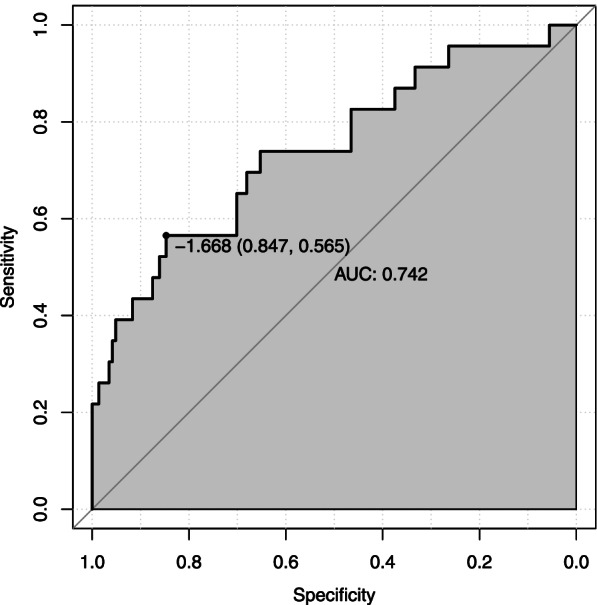
ROC curve of the nomogram model with the validation set

**Fig. 5:**
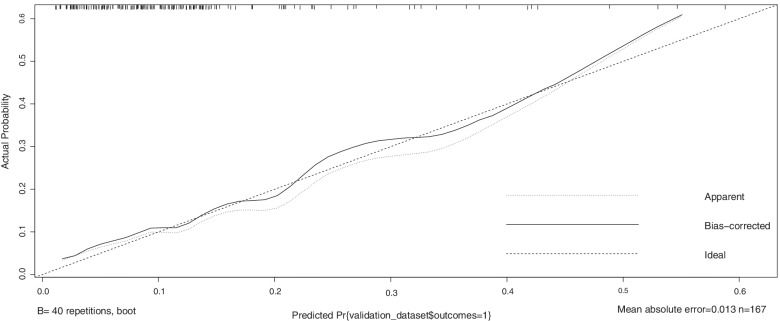
Calibration curve of the nomogram model with the validation set

External verification of the model was performed with the validation set. The results yielded an AUC of 0.742 *(95% CI*: 0.565 ~ 0.847) (Fig. 4). Moreover, the calibration chart showed that the model prediction curve fit well with the ideal curve (Fig. 5). The AUCs with both the training set and verification set were greater than 0.7. This shows that the model has good prediction ability.

## Discussion

Related studies have shown that older age is an important influencing factor of PE [22]. With increasing age, older pregnant women are more likely to have complications during pregnancy because of lower level of adaptability. Older pregnant women with PE are more likely to have adverse outcomes, which poses a serious challenge to maternal and infant health [23]. Due to the rapid development of China’s economy, China’s national conditions have changed significantly. Young people today focus more on their studies and work, delaying attempts at pregnancy. With the implementation of the “three-child policy”, some multiparous women have chosen to have two or more children, and most of them are over 35 years old. These two factors together have led to an increase in the age of pregnant women in China, which in turn leads to an increase in the probability of suffering from various complications during pregnancy. Therefore, it is necessary to establish a disease prediction model for this kind of pregnant woman that can not only improve maternal and infant health but also predict the possibility of adverse outcomes early to ensure timely implementation of effective treatment measures.

The occurrence of adverse outcomes in patients with PE is affected by many factors. We reviewed the medical records of 896 subjects through the hospital’s medical record management system. We selected scientifically relevant indicators that are easy and cost-effective to obtain in the routine diagnosis and treatment of pregnant women. The final results showed that platelet count (PLT), uric acid (UA), blood urea nitrogen (BUN), prothrombin time (PT) and lactate dehydrogenase (LDH) were independent influencing factors of adverse outcomes in advanced-age pregnant women with preterm PE. Our study showed that a long prothrombin time and low platelet count may increase the probability of an adverse outcome, which is consistent with previous studies [24]. A hypercoagulable state during pregnancy is an important physiological basis for PE. Patients with PE in a pathologically hypercoagulable state tend to develop thrombosis, which leads to insufficient placental perfusion and decreased placental function. All of these factors in turn lead to adverse outcomes, such as foetal growth restriction and placental abruption [25, 26]. Vascular endothelial cell injury, platelet adhesion and aggregation in patients with PE lead to vascular endothelial cell ischaemia and hypoxia, aggravate platelet destruction and lead to a decrease in PLT. This study found that higher levels of serum uric acid and blood urea nitrogen in patients with PE resulted in more obvious adverse effects on the outcome. This is consistent with the research of Hu et al. [27]. In patients with PE, the arterioles throughout the body are contracted, which induces ischaemia and anoxia in the foetus, while the uterus and placenta separate a large amount of lactate, excrete acidic substances, inhibit the excretion of uric acid by the kidney, and increase the level of serum uric acid, which leads to adverse outcomes [28]. PE patients are injured by ischaemia and hypoxia in various organs and tissues caused by systemic arteriole spasm, in which the damage to the liver and kidney is the most obvious, but changes in renal function are more obvious than those in liver function. In patients with PE, extensive spasm of the renal arterioles leads to glomerular swelling and a decrease in renal blood flow and in the glomerular filtration rate, which leads to a decrease in renal excretion function, hinders the clearance and excretion of UA, BUN and other metabolites in the blood, and occludes blood vessels, resulting in a significant increase in serum level [29]. Finally, we also found that LDH was related to the occurrence of adverse outcomes. As PE progresses, vascular wall tension and vascular endothelial cell injury and permeability increase. This leads to leakage of a large amount of protein and fluid into the tissue space, resulting in a decrease in plasma protein levels, an increase in blood viscosity, an imbalance in blood oxygen supply, and an increase in LDH levels in the body [30]. This study also confirmed that the pathogenesis of PE is the result of the joint action of multiple systems and multiple pathways. Based on the above indices, we can predict the probability of adverse outcomes in PE from indices of renal function and blood coagulation.

Previously, scholars developed many methods to predict adverse outcomes in patients with PE, but these methods have not been widely used. Some scholars chose to use maternal demographic data and clinical history, but due to ethnic limitations and individual differences, this method had low predictive ability. Some scholars have studied the impact of special biomarkers, such as soluble fms-like tyrosine kinase 1 (sFlt-1) and placental growth factor (PlGF), on the adverse outcomes of PE [31]. However, prediction effects based on one or several biomarkers are poor, and considering the cost, this method may not be suitable for the general population. At present, some scholars are committed to combining maternal demographic data and medical history information as well as certain auxiliary examinations to predict adverse outcomes. Liao et al. [32] established a risk prediction model for adverse outcomes in PE based on the statistical method of logistic regression, but it is inconvenient for doctors to apply a model with a large number of variables, and the model takes into account the entire population of PE and does not distinguish between different types of PE. LSaleh et al. [33] e stablished a prediction model of adverse outcomes by combining maternal factors and special biomarkers, but the sample size of this study and the range of sample collection were small, and the representativeness of the results was poor. This study reviewed a large amount of routine pregnancy examination data from older-age pregnant women with PE in China, including demographic data and basic medical history data as well as laboratory examination and ultrasound-assisted examination data. This kind of data is easily obtained in clinical applications. The nomogram prediction model of adverse outcomes in PE constructed in this study showed good predictive and discriminative ability, which indicates that the model can accurately predict the risk of adverse outcomes in advanced-age pregnant women with preterm PE in China and is suitable for the Chinese population. The prediction model, when presented in the form of a nomogram, is more intuitive and convenient for clinical medical staff to use [34]. The model provides corresponding guidance and serves as a basis for preventing adverse outcomes and improving clinical treatment and maternal and infant prognosis. Medical staff members can evaluate the risk of disease quickly based on changes in patients’ condition to improve the treatment plan and reduce the occurrence of adverse outcomes. Clinicians can judge the focus of treatment according to the score of each factor in the model, and specify an individual treatment plan for each patient, so as to reduce the harm of the disease and ensure the maximization of maternal and infant health.

### Limitations of the study

This study had four limitations. First, the data used to create the nomogram prediction model were obtained from a single centre, which may have induced bias. We will continue to carry out relevant research in the future to expand the research scope and sample size to enhance the predictive ability of the model. Second, this was a retrospective study, and the indicators included in the hospital’s routine examination were all included, so it was impossible to explore the predictive ability of other factors in this model. We intend to conduct prospective studies in the future to expand the collection of indicators. Third, this study was carried out in China, and the medical records used for model construction were all from pregnant Chinese women. Due to differences among regions and races, the applicability of the model to other countries needs to be further verified. Finally, The advanced-age pregnant women with preterm preeclampsia is a very high-risk group which is prone to adverse outcomes. Therefore, in addition to the meaningful factors in the results of this study, there may be more meaningful indicators. We will continue to explore more meaningful factors in future studies and verify the factors included in this study.

## Conclusions

In summary, a nomogram model for predicting adverse outcomes in advanced-age pregnant women with preterm preeclampsia based on PLT, UA, BUN, PT and LDH was constructed. The nomogram prediction model performed well and accurately predicted the risk of adverse outcomes in this population. Additional, according to the results of the study, the occurrence of adverse outcomes is highly related to the performance of the blood coagulation system and to renal function, which provides a direction for follow-up research. The model is simple to use and convenient for clinical medical staff for making dynamic evaluations according to the condition of the patient. The model provides corresponding guidance and serves as a basis for preventing adverse outcomes and improving clinical treatment and maternal and infant prognosis.

## Data Availability

The datasets used and analyzed during the current study are available from the corresponding author on reasonable request.
